# Divergent Delivery and Expression Kinetics of Lipid and Polymeric Nanoparticles across mRNA Modalities

**DOI:** 10.1002/advs.202508907

**Published:** 2025-07-12

**Authors:** Irafasha C. Casmil, Josh J. Friesen, Nuthan V. Bathula, Anneke Strumpel, Chia Hao Ho, Ilana Guez, Kristen Y. S. Kong, Andrew J. Varley, Shigeki J. Miyake‐Stoner, Parinaz Aliahmad, Nathaniel S. Wang, Andrew J. Geall, Anna K. Blakney

**Affiliations:** ^1^ Michael Smith Laboratories University of British Columbia Vancouver V6T 1Z4 Canada; ^2^ School of Biomedical Engineering University of British Columbia Vancouver V6T 2A1 Canada; ^3^ RWTH Aachen University Templergraben 55 52062 Aachen Germany; ^4^ RNA & Formulation Core University of British Columbia Vancouver V6T 1Z4 Canada; ^5^ Replicate Bioscience Inc San Diego CA 92121 USA

**Keywords:** circular mRNA, expression, linear mRNA, lipid nanoparticles, pABOL, self‐amplifying mRNA

## Abstract

Messenger ribonucleic acid (mRNA)‐based therapies, including conventional linear mRNA (linRNA), circular RNA (circRNA), and self‐amplifying RNA (saRNA), are being developed not only for vaccination but also for protein replacement, gene editing, and regenerative medicine. However, these mRNA modalities differ in structure and function, and their interactions with current non‐viral delivery systems influence their therapeutic efficacy. Here, the in vivo expression kinetics of linRNA, circRNA, and saRNA delivered via lipid nanoparticles (LNPs) or bioreducible poly(cystamine bisacrylamide‐co‐4‐amino‐1‐butanol) (pABOL) polymer are systematically evaluated. At 0.5 µg, Venezuelan equine encephalitis virus (VEEV)‐based saRNA resulted in higher total luciferase expression than 5 µg of linRNA or circRNA highlighting its superior potency. LNPs significantly enhanced expression of non‐amplifying mRNAs compared to pABOL, whereas pABOL delivery of saRNA yielded a ∼2‐fold improvement over LNPs. Furthermore, saRNAs derived from New World alphaviruses expressed 2–6 times more protein than Old World saRNAs when delivered with LNPs; these differences are not observed with pABOL. These findings demonstrate that mRNA modality, saRNA genotype, and delivery platform interact to determine therapeutic protein output. This study provides actionable insights for optimizing mRNA‐based therapeutics across diverse clinical applications.

## Graphical abstract

Self‐amplifying (saRNA), linear (linRNA), and circular (circRNA) mRNAs are compared under standardized conditions using lipid nanoparticles (LNPs) and pABOL polymer. saRNA achieved superior expression, while linRNA and circRNA performance varied based on untranslated region elements and delivery method. Differences between alphavirus saRNAs are enhanced by LNPs but attenuated with pABOL. These findings support rational selection of RNA modality–delivery platform combinations.

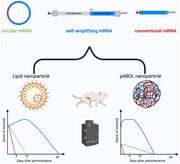

## 1 Introduction

The 2019 Coronavirus disease (COVID‐19) pandemic and the global race to develop vaccines resulted in the “RNAissance” – a renaissance in genetic medicines fueled by significant milestones in messenger ribonucleic acid (mRNA).^[^ ^1^ ^]^ mRNA‐based medicines function in the cytosol of mammalian cells and may be designed with or without self‐amplifying properties, depending on whether they encode a viral replicase to enable intracellular RNA amplification. Non‐amplifying mRNA exist as either conventional linear (linRNA) or circular (circRNA) form,^[^ ^2^ ^]^ while replicating mRNA, commonly referred to as self‐amplifying RNA (saRNA), is derived from alphaviruses.^[^ ^3^ ^]^ Much of the initial clinical success of mRNA technologies has focused on vaccines, particularly linRNA‐based products such as Comirnaty, Spikevax and mResvia, as well as the saRNA‐based vaccine Kostaive.^[^ ^4^, ^5^ ^]^ There is growing interest in expanding application of these platforms beyond immunization, particularly protein replacement therapies for genetic diseases, regenerative medicine and launching of gene editing tools.^[^ ^6^ ^]^ Due to the instability of RNA molecules and susceptibility to extracellular nucleases, advancement in lipid and polymeric delivery of RNA has been an integral and central research field in the RNAissance.^[^ ^7^ ^]^ Therefore, understanding how the mRNA modality and delivery vehicle influence kinetics is critical for the advancement of next‐generation mRNA‐based therapeutics.

The structural and functional characteristics of each mRNA modality influence their stability, translational capacity, and suitability for therapeutic protein expression.^[^ ^8^ ^]^ linRNA is designed with a 5′ cap, an open reading frame of the gene of interest (GOI), or antigen, flanked by untranslated regions (UTR) and a poly‐adenosine tail. Upon delivery into the cytosol, linRNA transcripts undergo cap‐dependent translation of the GOI but their expression is short‐lived primarily due to degradation by exonucleases and cellular turnover.^[^ ^9^ ^]^ To date, only three conventional mRNA‐based vaccines are approved: Comirnaty and Spikevax for COVID‐19, and mResvia for respiratory syncytial virus.^[^ ^10^, ^11^, ^12^ ^]^ Although linRNA COVID‐19 vaccines initially demonstrated high efficacy, antibody titres declined within six months and resulted in lower immunogenicity against emerging variants.^[^ ^13^ ^]^ Similarly, clinical data from the Phase 3 mResvia trial show that efficacy decreased from 78.7% at 3.7 months to 62.5% at 8.6 months and further declined to 50.3% at 18 months post‐vaccination.^[^ ^14^ ^]^ This waning immunity may, in part, reflect the short duration of antigen expression inherent to linRNA platforms, which could limit the development of durable germinal center responses and long‐lived immune memory.^[^ ^15^ ^]^ Furthermore, Kormann et al. demonstrated in a mouse model of congenital surfactant protein B (SP‐B) deficiency that two doses of modified SP‐B mRNA, delivered intratracheally, extended survival for only a few days. In contrast, biweekly aerosol administration over four consecutive weeks restored SP‐B expression to near‐physiological levels and rescued mice throughout the 28‐day study period.^[^ ^16^ ^]^ These findings highlight the critical role of sustained protein expression in achieving prolonged therapeutic and immunological effects.

circRNA has received considerable interest as an alternative to linRNA due to its relatively higher stability.^[^ ^17^ ^]^ The closed ring conformation of circRNA protects it from exonuclease degradation, resulting in a median half‐life ∼2.5 times longer than that of linRNA in mammalian cells.^[^ ^18^ ^]^ Synthetic circRNA for protein expression are engineered to contain internal ribosomal entry site (IRES) elements for cap‐independent translation of the downstream GOI. Chen et al. demonstrated in mice that an optimized design of circRNA encoding human erythropoietin (hEPO) resulted in increased and consistent plasma levels of hEPO for 96 h while its linRNA counterpart rapidly declined within 48 h.^[^ ^19^ ^]^ Similarly, mice vaccination studies with circRNA or nucleoside‐modified linRNA encoding a modified SARS‐CoV‐2 antigen showed comparable total immunoglobulin G (IgG) titres.^[^ ^20^ ^]^ These findings indicate that circRNA extends the duration of protein expression relative to linRNA. To further extend the duration and magnitude of protein expression, saRNA platforms have been developed, leveraging alphaviral replicase machinery to enable intracellular mRNA replication.

saRNA is structurally similar to linRNA but encodes an additional open reading frame of an alphavirus replicase.^[^ ^21^ ^]^ saRNA is predominantly derived from Sindbis, Semliki Forest and Venezuelan equine encephalitis viruses (VEEV), with the latter being the most clinically advanced. Kostaive, the first saRNA vaccine approved against SARS‐CoV‐2, is administered at 5 µg to patients, compared to the 30 or 100 µg of Spikevax and Comirnaty, respectively.^[^ ^10^, ^11^, ^22^ ^]^ saRNA vaccination at significantly lower doses indicates their potential for cost‐effective scale‐up and distribution. Beyond vaccines, the duration and kinetics of saRNA expression were modulated by using other alphaviral species, with some vectors resulting in reporter protein expression for over 25 days in mice.^[^ ^23^ ^]^ Despite advances in saRNA vector design, efficient delivery is essential for maximizing therapeutic protein expression.

The advancement of mRNA medicines has heavily relied on the successful delivery of the highly anionic RNA molecule across cellular membranes and facilitating its cytosolic release for protein expression. Delivery of various RNA modalities is facilitated by lipid (LNPs) and polymeric nanoparticles (PNPs). To date, LNPs have been utilized in four out of the five clinically approved mRNA or saRNA vaccines, underscoring their clinical maturity and translatability.^[^ ^10^, ^11^, ^22^, ^24^ ^]^ LNPs are a versatile platform that can be reconfigured for different mRNA payloads, but their utility is limited due to increased reactogenicity and systemic biodistribution even in the case of local administration.^[^ ^25^, ^26^ ^]^ Alternatively, cationic polymers complexed with mRNA modalities enable delivery into the cell, albeit at lower transfection efficiencies relative to LNPs.^[^ ^27^ ^]^ Bioreducible poly(cystamine bisacrylamide‐co‐4‐amino‐1‐butanol) (pABOL) has emerged as a promising polymeric carrier, demonstrating preclinical delivery of plasmid deoxyribonucleic acid (pDNA) and saRNA in mice.^[^ ^28^, ^29^ ^]^ Comparative studies evaluating saRNA formulated with either LNPs or pABOL highlighted key differences in delivery efficiency, protein expression kinetics, and innate immune activation profiles. Notably, pABOL‐mediated saRNA delivery has been shown to elicit higher local protein expression following intramuscular injection compared to LNPs at equivalent RNA doses, while simultaneously inducing lower levels of acute inflammatory cytokines such as interleukin‐6 and interferon‐beta.^[^ ^26^, ^30^ ^]^ In addition to these innate immune differences, Blakney et al. demonstrated that pABOL‐saRNA vaccines resulted in lower humoral and cellular immunity against an influenza virus antigen than LNPs.^[^ ^30^ ^]^ These comparative findings underscore the importance of aligning mRNA modality with an appropriate delivery system to optimize therapeutic outcomes.

Building on previous studies, we investigated the delivery of conventional linear mRNA, circular RNA and saRNA using both pABOL PNPs and LNPs, herein. Our primary goal was to determine whether PNPs would enhance the expression of replication‐incompetent mRNA (linRNA and circRNA) as has been observed for saRNA PNPs. Furthermore, we aimed to explore whether the delivery vehicle would enhance expression from both Old and New World alphavirus‐derived saRNA vectors. This allowed us to systematically evaluate how delivery modality influences both the magnitude and duration of protein expression, providing new insights into the interplay between saRNA vector design and nanoparticle delivery systems.

## 2 Results

### 2.1 Design and Characterization of mRNA modalities and delivery modules

**Figure 1:**
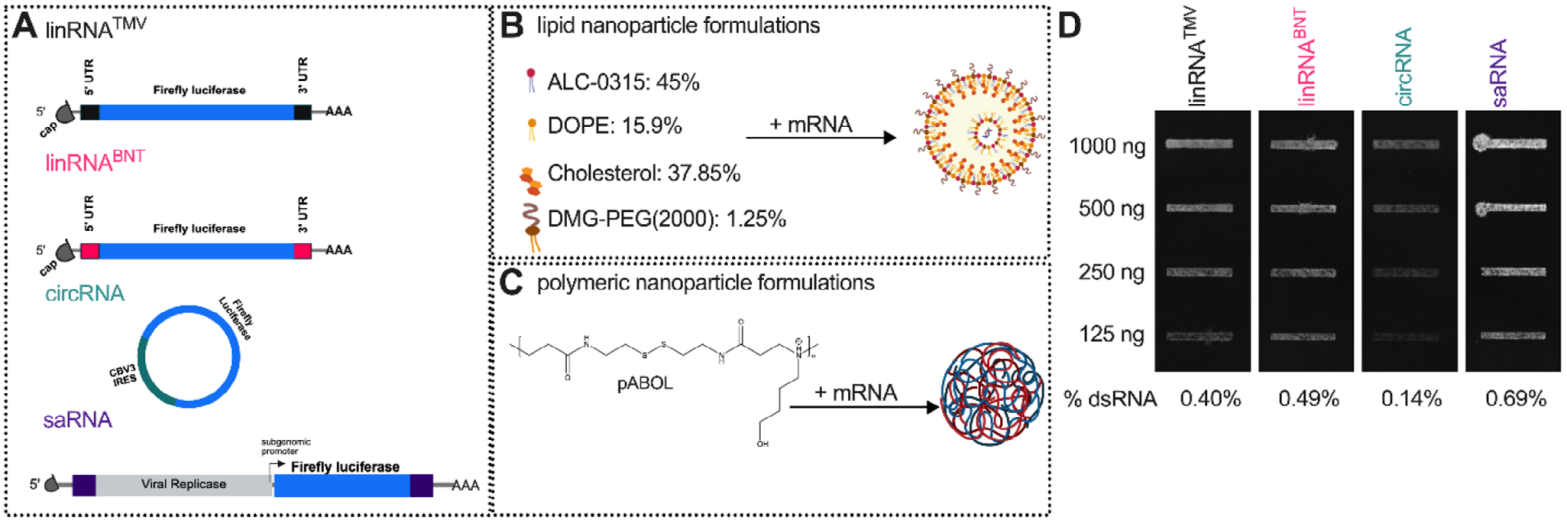
Characterization of mRNA modalities and delivery modules. A) Illustration of the structural elements of the in vitro transcribed linear, circular and self‐amplifying mRNA. B) Lipid mixture composition formulated with mRNA modalities using microfluidic mixing to generate LNPs. C) Chemical structure of pABOL polymer complexed with mRNA modalities to generate PNPs. D) Dot blot image of double‐stranded RNA content in a serial dilution of mRNA modalities used.

The structural elements of the three mRNA modalities (linear – linRNA; circular – circRNA; self‐amplifying – saRNA) encoding firefly luciferase (Fluc) are illustrated in **Figure** **1**A. These mRNA modalities were formulated into LNPs (Figure 1B) or PNPs using pABOL (Figure 1C). Two linRNA transcripts differing in UTRs were assessed. linRNA^TM^ ^V^ contained 5′ and 3′ UTRs obtained from the Tobacco mosaic virus (TMV), a positive‐sense plant RNA virus of the *Tobamovirus* genus.^[^ ^31^ ^]^ Phylogenetic clustering based on the RNA‐dependent RNA polymerase of other positive‐sense, single‐stranded RNA viruses categorizes TMV as part of the alphavirus‐like supergroup.^[^ ^32^ ^]^ In contrast, the UTRs of linRNA^BNT^ were based on the Comirnaty SARS‐CoV‐2 vaccine,^[^ ^10^ ^]^ in which the 5′ was obtained from the human α‐globin gene, and the 3′ UTR contained optimized segments of the human mitochondrial 12S ribosomal RNA (mtRNR1) and the AES/TLE5 gene.^[^ ^33^, ^34^ ^]^ The circular RNA was generated using a permuted intron‐exon system for circularization.^[^ ^19^, ^35^ ^]^ For translation initiation in the circRNA, the coxsackievirus B3 IRES was chosen owing to evidence demonstrating that these elements resulted in higher expression of luciferase in vitro and in vivo compared to other viral IRES elements.^[^ ^19^, ^36^ ^]^ The control saRNA vector was derived from the TC83 variant of VEEV and was previously used clinically as a rabies vaccine platform (NCT06048770).^[^ ^37^ ^]^ Considering that the linRNAs were generated using N1‐methylpseudouridine while circRNA and saRNA were synthesized using standard nucleotides, we quantified the presence of double‐stranded RNA (dsRNA) with an anti‐dsRNA antibody (Figure 1D). Of the loaded 125 nanograms, saRNA had the highest dsRNA content of ≈0.69% of the loaded RNA while circRNA had the least amount. Both linRNAs had relatively similar levels of dsRNA content. Notably, all mRNA modalities contained less than 1% dsRNA content. To ensure comparable delivery conditions across mRNA modalities, we next characterized the physicochemical properties of the formulated lipid and polymeric nanoparticles.

**Figure 2:**
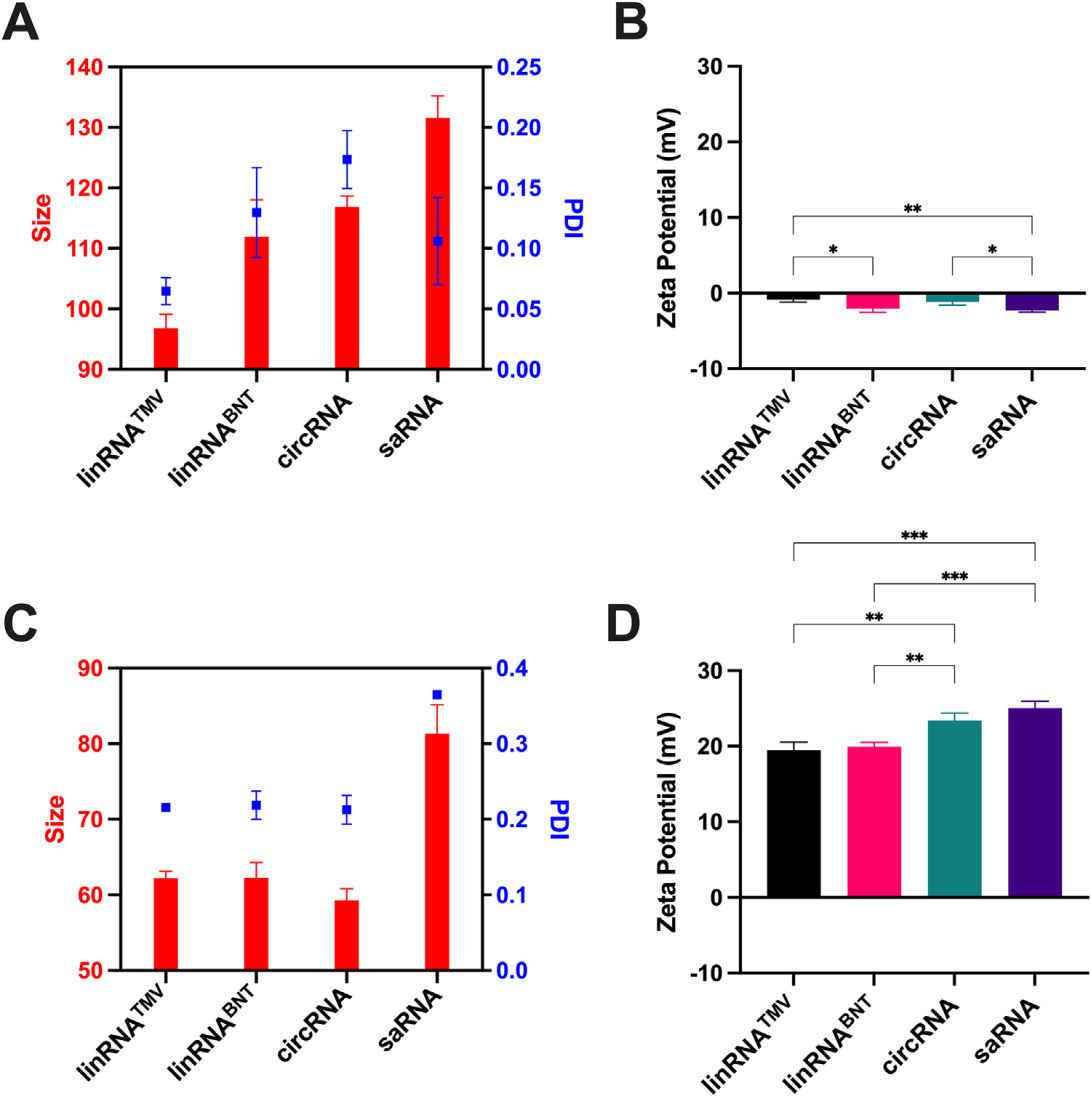
Physicochemical properties of mRNA nanoparticles. A) Average zeta size in nanometers and polydispersity index of LNPs. B) Zeta potential in millivolts of LNPs. C) Zeta size in nanometers and polydispersity index of PNPs. D) Zeta potential in millivolts of PNPs. Data shown are mean values ± standard deviation from n = 3 technical replicates compared using One‐way ANOVA followed by Tukey's multiple comparison test. ^*^ *p* < 0.05; ^**^ *p* < 0.01; ^***^ *p* < 0.001; ^****^ *p* < 0.0001.

For uniformity, all mRNA modalities were encapsulated in LNPs or complexed with the pABOL copolymer under standardized formulation conditions. LNP formulations were prepared as previously described for optimal physicochemical attributes and reduced cellular activation, using a nitrogen‐to‐phosphate (N/P) ratio of 10.^[^ ^38^ ^]^ All LNP formulations had >95% encapsulation efficiency. The zeta‐average hydrodynamic size of the LNP‐mRNA formulations was significantly different (ANOVA, P < 0.0001) except between linRNA^BNT^ and circRNA (**Figure** **2**A). All LNP‐mRNA formulations exhibited near‐neutral zeta potential (Figure 2B). Similarly, all mRNA modalities were complexed with pABOL at a 45:1 weight‐to‐weight (w/w) ratio.^[^ ^26^ ^]^ pABOL‐saRNA polymeric nanoparticles (PNP) had significantly larger particles than other polyplexes (Figure 2C). All pABOL‐mRNA formulations displayed a positive surface charge, with zeta potentials exceeding 19 mV (Figure 2D). Having characterized the physicochemical profiles of LNPs and pABOL‐based polyplexes for each mRNA modality, we next evaluated their resulting in vivo protein expression kinetics.

### 2.2 PNP and LNP Delivery of mRNA Modalities Result in Different Protein Expression Kinetics

#### 2.2.1 LNP Delivery Accentuates the Total Transgene Expression of saRNA, circRNA and linRNA

**Figure 3:**
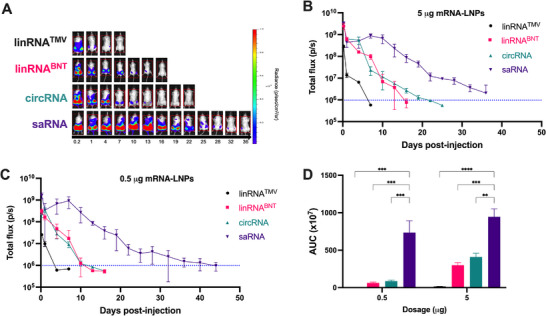
Luciferase expression after LNP delivery of mRNA modalities. A) Bioluminescence images of mice after intramuscular injection of 5 µg of linRNA^TMV^ (black), linRNA^BNT^ (magenta), circRNA (turquoise) and VEEV‐saRNA (purple). B) Total flux detected on days indicated after administration of 5 µg or C) 0.5 µg of LNP‐mRNA. Data shown are mean values ± standard deviation from n = 3 biological replicates. D) Area under the curve of total flux curves for the duration of the study. Data shown are mean ± standard error of mean (SEM) compared using two‐way ANOVA followed by Tukey's multiple comparison test. ^**^ *p* < 0.01; ^***^ *p* < 0.001; ^****^ *p* < 0.0001.

We next evaluated the impact of LNP and PNP delivery platforms on mRNA expression kinetics and magnitude after intramuscular injections. Bioluminescence imaging was performed longitudinally to assess total luciferase expression in female BALB/c mice over time. LNPs containing 0.5 or 5 µg of linRNA^TM^ ^V^, linRNA^BNT^, circRNA and saRNA resulted in substantial differences between the replicating and non‐replicating modalities (**Figure** **3**A). After delivery with LNPs at high (Figure 3B) and low doses (Figure 3C), the non‐replicating mRNA modalities showed peak expression 4 hours (h) after injection and decreased thereafter. Expression from linRNA^TM^ ^V^ diminished to baseline levels by days 4 – 7, linRNA^BNT^ by days 11–16 and circRNA by days 12–22 post‐injection, as determined by total flux analysis. However, visible bioluminescence was largely absent at later time points, suggesting that while residual expression persisted above background, it was likely biologically minimal. In contrast, saRNA exhibited an initial peak at 4 h post‐injection, followed by a second local maximum around day 7 for both doses. saRNA expression declined to baseline between days 36 and 44, approximately 2–3 weeks after the non‐amplifying mRNA groups returned to baseline levels.

A dose‐dependent increase in luciferase expression was observed for all mRNA modalities. Compared to 0.5 µg of LNP‐mRNA, administration of 5 µg of linRNA^V^, linRNA^BNT^, circRNA and saRNA resulted in 5.8‐, 4.8‐, 4.7‐, and 1.3‐fold higher total bioluminescence, respectively, as quantified by the area under the curve of total flux (Figure 3D). Notably, a smaller difference in total expression was observed between the higher and lower dose saRNA groups. When compared to non‐replicating mRNA modalities, saRNA luciferase expression after LNP administration was 60–272 times higher than linRNA^V^, 3.2‐11 fold higher than linRNA^BNT^, and 2.3‐8 times higher than circRNA, with the largest difference observed at the low dose. Together, these results demonstrate that LNP delivery of linRNA facilitated rapid but short‐lived protein production while circRNA offered a moderate balance between expression duration and magnitude. In contrast, LNP‐saRNA provided superior durability and protein output, even at lower RNA doses, underscoring its potential for therapeutic applications requiring sustained protein replacement over extended periods.

#### 2.2.2 PNP Delivery Augments saRNA Expression as well as Ablates linRNA and circRNA Expression

**Figure 4:**
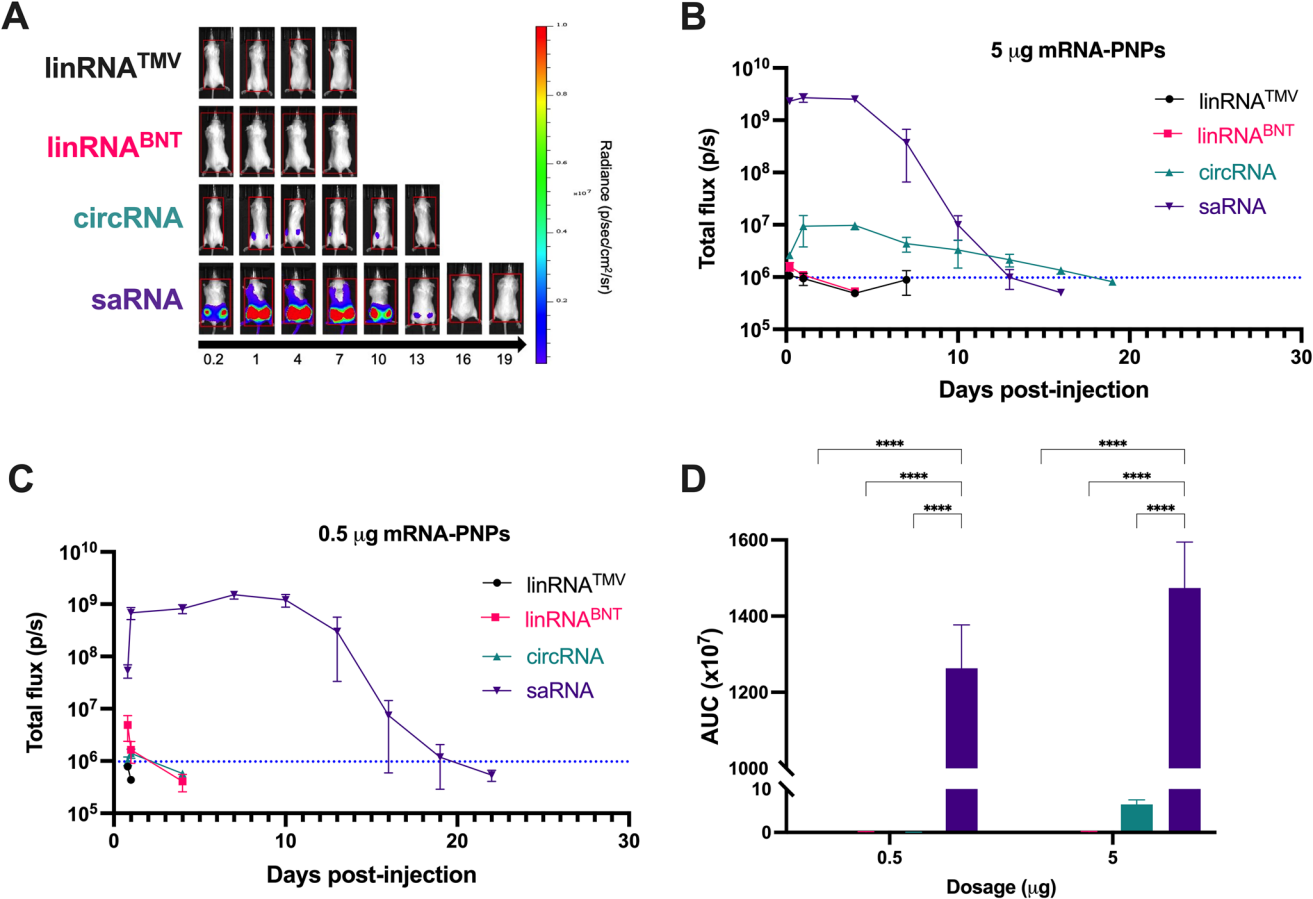
Luciferase expression after PNP delivery of mRNA modalities. A) Bioluminescence images of mice after intramuscular injection of 5 µg of linRNA^TMV^ (black), linRNA^BNT^ (magenta), circRNA (turquoise) and VEEV‐saRNA (purple). B) Total flux detected on days indicated after administration of 5 µg or C) 0.5 µg of pABOL‐mRNA. Data shown are mean values ± standard deviation from n = 3 biological replicates. D) Area under the curve of total flux curves for the duration of the study. Data shown are mean ± standard error of mean (SEM) compared using two‐way ANOVA followed by Tukey's multiple comparison test. ^****^ *p* < 0.0001.

To determine whether PNP delivery supports efficient protein expression across different mRNA modalities, we evaluated the in vivo expression kinetics of linRNA, circRNA, and saRNA formulated into pABOL PNPs. Given that pABOL has predominantly been studied for the delivery of larger nucleic acids such as saRNA and plasmid DNA,^[^ ^39^ ^]^ we sought to assess its capacity to deliver non‐replicating linRNA and circRNA transcripts. Interestingly, PNP delivery of linRNA^V^ and linRNA^BNT^ at both high (**Figure** **4**A,B) and low (Figure 4C) doses resulted in detectable luciferase expression above the PBS background only at 4 and 24 h post‐injection, respectively. Beyond day 1, bioluminescence for the linRNA‐PNP groups was not detectable. Administration of 5 µg of circRNA PNPs resulted in luciferase expression at 4 h, reached peak expression at 24 h, remained at peak levels for 4 days, and then gradually declined to baseline levels by the 16th day. At 0.5 µg, luciferase expression from the pABOL‐circRNA group was only detectable 1 day post‐injection.

Conversely, pABOL delivery of saRNA resulted in higher luciferase expression, followed by a rapid decline on days 7 and 10 for the 5 and 0.5 µg groups, respectively. Bioluminescence after administration of 0.5 and 5 µg of saRNA‐PNP declined to baseline levels on days 20 and 13, respectively. 5 µg of pABOL delivery of linRNA^V^, linRNA^BNT^, circRNA and saRNA resulted in 11.7‐, 1.2‐, 96.2‐ and 1.2‐fold higher luciferase expression compared to the 0.5 µg groups (Figure 4D). Notably, 5 µg of saRNA PNPs resulted in 1.7 × 10^5^, 8.8 × 10^3^ and 2.37 × 10^2^‐fold higher expression than PNPs of linRNA^V^, linRNA^BNT^ and circRNA, respectively. These findings demonstrated that pABOL delivery is most effective for large, replicating saRNA cargos, while delivery of non‐replicating linRNA and circRNA may require polymer optimization to achieve efficient delivery in vivo.

#### 2.2.3 LNPs Enable Robust Expression of mRNA Modalities Compared to PNP Delivery

To assess the relative efficiency of LNP versus PNP delivery across mRNA modalities, we compared in vivo luciferase expression following intramuscular administration of linRNA, circRNA, or saRNA, each formulated with either delivery system. At a dose of 5 µg, LNP delivery of linRNA^V^, linRNA^BNT^, and circRNA (Figure 3A–D) resulted in 184‐, 180‐, and 63‐fold higher luciferase expression, respectively, compared to PNP delivery (Figure 4A–D). Given that pABOL‐mRNA PNPs were formulated at a 45:1 weight‐to‐weight (w/w) ratio, an optimized condition previously reported for saRNA delivery, we next evaluated whether altering the polymer‐to‐RNA ratio could improve linRNA or circRNA expression. Adjusting the w/w ratio of pABOL to RNA, either increasing or decreasing polymer amounts, did not enhance luciferase expression from linRNA^V^, linRNA^BNT^, or circRNA polyplexes (Figure S1, Supporting Information). Interestingly, PNP delivery of saRNA (Figure 4D) resulted in ≈2‐fold higher luciferase expression compared to LNP‐saRNA delivery (Figure 3D), consistent with enhanced local expression driven by the cationic nature of pABOL polyplexes.

In summary, LNP formulations used in this study demonstrated high delivery efficiency and enabled robust protein expression for all three mRNA modalities (linRNA, circRNA and saRNA). In contrast, pABOL, in its current optimized form, enabled functional delivery of large saRNA as evidenced by sustained protein expression but was suboptimal for non‐amplifying mRNA modalities. These findings highlight delivery modality‐specific considerations for different mRNA platforms and suggest that future polymer development will require tuning beyond simple formulation parameters to improve the delivery of smaller RNA species.

### 2.3 Influence of Delivery System and Alphaviral Genotype of saRNA Expression

**Figure 5:**
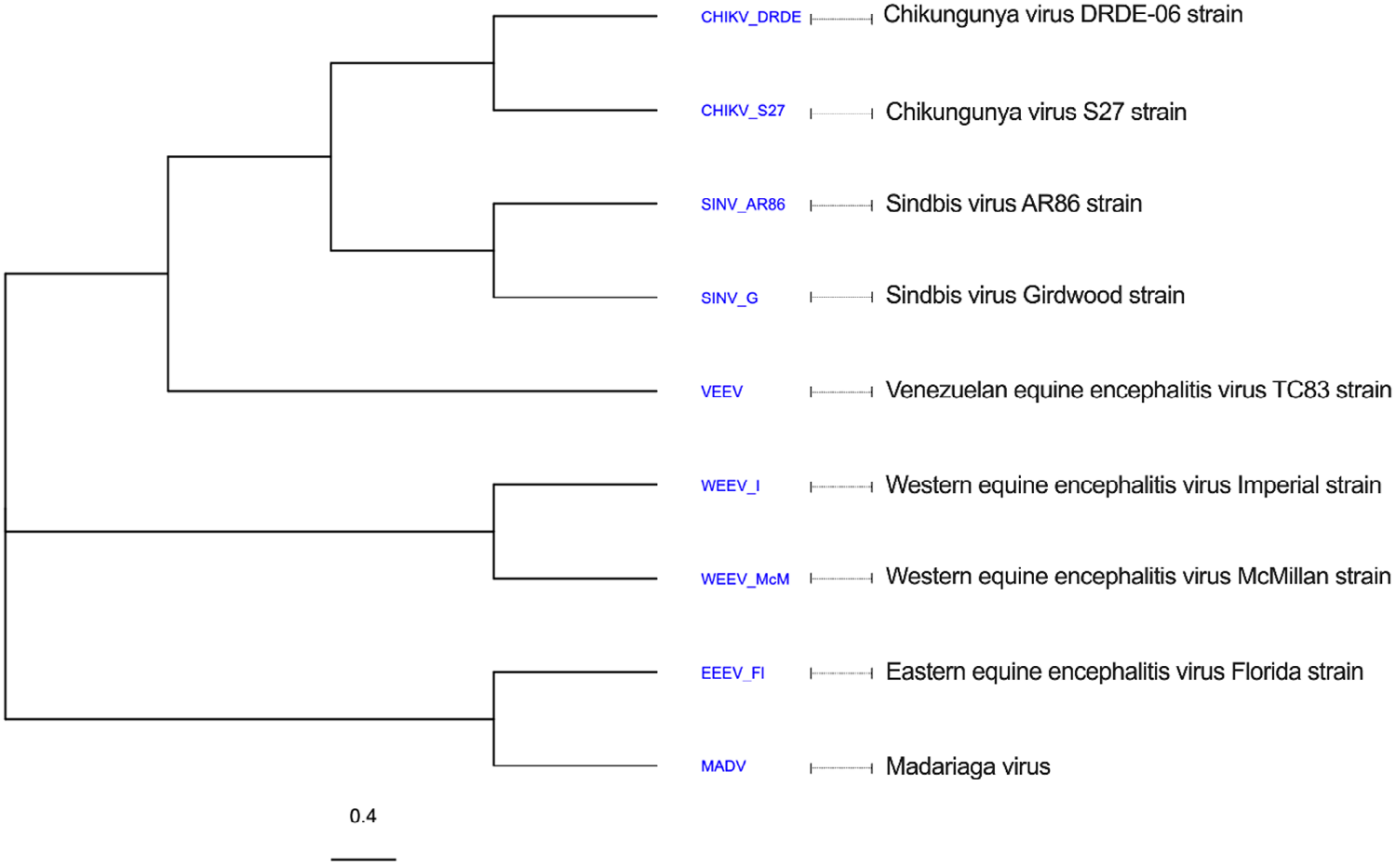
Phylogenetic analysis of alphavirus‐derived saRNA vectors evaluated. Phylogenetic tree generated using neighbor‐joining method based on the amino acid sequences of the non‐structural protein 4. Branch lengths represent evolutionary distances.

Given the observed advantage of pABOL and LNPs for delivering large, self‐amplifying RNA compared to non‐replicating mRNA, we next sought to determine whether this delivery advantage is preserved across saRNA vectors derived from different alphavirus species. While VEEV‐based saRNA currently represents the most clinically advanced platform, previous studies have demonstrated that other alphaviruses can also be adapted as saRNA vectors with varying replication kinetics and host cell interactions.^[^ ^23^, ^40^ ^]^ To address this, we evaluated a panel of eight saRNA vectors derived from four New World and four Old World alphaviruses (Table S1, Supporting Information), alongside VEEV as a reference control (**Figure** **5**). Phylogenetic analysis of the non‐structural protein 4 (nsP4) region, using the neighbor‐joining method, revealed a tree topology placing VEEV centrally within the alphavirus family, reflecting its evolutionary divergence and widespread use in saRNA technologies. The phylogenetic topology and branch lengths were consistent with established alphavirus evolutionary relationships, with a scale bar indicating 0.4 substitutions per site. The phylogenetic tree revealed distinct evolutionary groupings. The rapid outbreak DRDE‐06 and prototypical S27 Chikungunya virus (CHIKV) strains clustered closely together, as did the neurovirulent AR86 and avirulent Girdwood Sindbis virus (SINV) strains. VEEV branched separately but remained more closely related to CHIKV and SINV than to the Western equine encephalitis viruses (WEEV Imperial and McMillan strains) and Eastern equine encephalitis virus (EEEV Florida 93–939 strain). The WEEV strains formed a distinct subclade, while EEEV and Madariaga virus (MADV) grouped together, reflecting their greater evolutionary divergence. All saRNA vectors were formulated into LNPs or pABOL polyplexes using standardized conditions described in the Experimental section. Notably, all LNP‐saRNA^X^ and pABOL‐saRNA^X^ formulations, where X denotes the alphavirus source, exhibited comparable physicochemical properties (Figure S3, Supporting Information). Although the saRNA constructs varied modestly in length, ranging from 9614 to 9876 nucleotides, equal mass dosing at 5 µg per injection resulted in highly similar molar quantities across all vectors, estimated at 1.60 ± 0.03 × 10^−12^ molecules (Table S2, Supporting Information). This uniformity in RNA copy numbers ensured that observed differences in in vivo expression kinetics primarily reflect vector‐intrinsic properties rather than discrepancies in administered saRNA dose.

#### 2.3.1 New World Alphavirus‐Derived saRNA Vectors Exhibit Superior and Prolonged Expression following LNP Delivery

**Figure 6:**
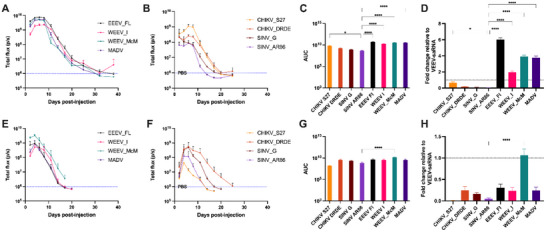
LNP and PNP delivery of genotypically distinct saRNA vectors. A) Total flux detected on days indicated after administration of LNPs containing 5 µg of New World and B) Old World alphaviral saRNA vectors. C) Area under the curve of total flux curves shown in A and B. D) Total expression relative to control VEEV‐saRNA after LNP administration. E) Total flux detected on days indicated after administration of pABOL complexed with 5 µg of New World and F) Old World alphaviral saRNA vectors. (C) Area under the curve of total flux curves shown in E and F. (D) Total expression relative to control VEEV‐saRNA after PNP administration. For A, B, E and F, data shown are mean values ± standard deviation from n = 3 biological replicates. For C, D, G, and H, data shown are mean ± standard error of mean (SEM) compared using one‐way ANOVA followed by Dunnett's multiple comparison test. ^*^ *p* < 0.05; ^****^ *p* < 0.0001.

To investigate whether alphavirus species influence the expression kinetics of saRNA in vivo, we compared LNP delivery of 5 µg of saRNA vectors derived from New World and Old World alphaviruses. Consistent with the prolonged expression observed for VEEV, a New World alphavirus, LNP delivery of saRNA derived from EEEV, WEEV, and MADV also resulted in sustained luciferase expression lasting up to or slightly beyond 30 days (**Figure** **6**A). In contrast, none of the Old World saRNA‐LNP groups, including CHIKV and SINV, exhibited luciferase expression beyond 20 days post‐injection (Figure 6B). Statistical analysis comparing total luciferase expression (area under the curve, AUC) from all LNP‐saRNA^X^ groups relative to SINV AR86, a commonly used saRNA vector in preclinical studies, demonstrated that all New World‐derived saRNA vectors induced significantly higher expression (p < 0.05). Among the Old World vectors, only CHIKV S27 showed significantly higher expression relative to SINV_AR86 (Figure 6C). Moreover, when compared directly to LNP delivery of VEEV‐saRNA, saRNA derived from WEEV_I, MADV, WEEV_McM, and EEEV_Fl achieved 2‐, 3.8‐, 3.9‐, and 6‐fold higher cumulative luciferase expression, respectively (Figure 6D). Conversely, LNP delivery of Old World alphavirus‐derived saRNAs consistently resulted in lower total expression compared to VEEV, underscoring the influence of alphavirus genotype on saRNA‐driven protein expression in vivo.

#### 2.3.2 PNP Delivery of Alphavirus‐Derived saRNA Vectors Results in Uniform Expression Kinetics Independent of Viral Genotype

Given that LNP delivery accentuated differences in expression kinetics between New World and Old World alphavirus‐derived saRNA vectors, we investigated whether pABOL delivery would similarly preserve these genotype‐dependent differences or instead result in uniform expression profiles across saRNA backbones. PNP delivery of the New (Figure 6E) and Old World (Figure 6F) alphavirus‐derived saRNA vectors resulted in comparable luciferase expression kinetics. With the exception of CHIKV_DRDE that was detectable up to day 22 post‐injection, all other pABOL‐saRNA^X^ expression was cleared by day 16. Similar to VEEV‐saRNA expression after 5 µg PNP delivery, all other saRNA vectors resulted in peak expression for 4–7 days, after which it declined sharply. Notably, despite these subtle differences in decay kinetics, total luciferase expression (AUC) across most pABOL‐delivered saRNA vectors was comparable. When benchmarked against the commonly used SINV_AR86 saRNA, only WEEV_McM resulted in significantly higher total expression (Figure 6G). Furthermore, when compared directly to the VEEV‐saRNA control, the majority of alternative saRNA vectors demonstrated lower total expression with pABOL delivery, with the exception of WEEV‐derived constructs which achieved comparable or higher cumulative expression (Figure 6H). Collectively, these results indicate that pABOL delivery produced more uniform saRNA expression kinetics across diverse alphavirus backbones.

#### 2.3.3 Delivery Platform Influences Duration and Decay Rate of saRNA Expression Across Alphavirus Genotypes

To characterize the effect of LNP and pABOL delivery on saRNA expression kinetics, we analyzed peak expression, the percentage decline at days 14 and 21 from peak expression, decay slopes and the time to baseline following intramuscular injections (**Table** **1**). Decay slopes were calculated using linear regression analysis of total bioluminescent flux starting from the day each construct reached peak expression. Considering that the saRNAs displayed differences in expression magnitude, a complementary analysis based on the percent decline from peak expression over time was performed to facilitate a more equitable comparison of relative decay. LNP delivery of New World alphavirus‐derived saRNAs such as EEEV_Fl, WEEV_McM, WEEV_I, and VEEV exhibited substantially prolonged expression, with baseline clearance times ranging from 27 to 38 days despite steeper decay slopes. Of the New World saRNA vectors, VEEV and WEEV_I showed sustained expression levels at day 14, retaining 23–78% of their peak values. In contrast, Old World saRNA vectors declined more rapidly after the peak, with most reaching baseline by day 20, despite exhibiting milder decay slopes. On the other hand, pABOL delivery resulted in compressed decay kinetics and lower persistence of expression overall. All pABOl‐formulated saRNAs peaked by day 4, except SINV_G and CHIKV_DRDE, and declined by >92% from their peak expression by day 14. Notably, CHIKV_DRDE maintained ≈7% of its peak expression by day 14, exhibited the shallowest pABOL decay slope and one of the longest durations of pABOL‐enabled expression (22 days). Taken together, these results highlight that the decay kinetics of saRNA‐driven protein expression depend not only on the vector genotype but also on the delivery strategy. LNPs more clearly preserved genotype‐dependent differences, favoring extended expression of New World replicons, while pABOL delivery led to uniform and rapid signal clearance, suggesting delivery‐mediated compression of genotype‐specific expression profiles.

**Table 1:** Comparative summary of decay kinetics following delivery of 5 µg saRNA in LNPs and PNPs.

saRNA	LNPs				pABOL			
	Peak day	% decline on day 14 [mean±SEM]	Days to baseline	Decay slope × 10^7^ [total flux per day]	Peak day	% decline on day 14 [mean±SEM)]	Days to baseline	Decay slope × 10^7^ [total flux per day]
EEEV_Fl	6	96.98 ± 0.071	32	−14	4	99.83 ± 0.06	20	−3.6
WEEV_McM	4	99.15 ± 0.09	29	−12	4	99.46 ± 0.35	21	−12.2
VEEV	7	74.19 ± 9.34	30	−1.8	4	99.96 ± 0.01	13	−18
WEEV_I	8	89.72 ± 1.14	38	−5.5	4	99.62 ± 0.22	18	−3.4
CHIKV_DRDE	6	94.45 ± 1.19	20	−1.1	7	92.42 ± 5.57	22	−1.8
MADV	8	98.90 ± 0.03	27	−1.5	4	99.77 ± 0.11	16	−1.9
SINV_G	2	98.89 ± 0.46	19	−0.5	7	99.28 ± 0.14	17	−1
SINV_AR86	6	99.15 ± 0.23	18	−0.2	4	99.51 ± 0.36	16	−0.9
CHIKV_S27	6	99.04 ± 0.40	18	−4.1	4	98.58 ± 0.19	15	−0.1

## 3 Discussion

mRNA‐based therapeutics are expanding beyond vaccines into applications such as protein replacement, gene editing, and regenerative medicine. As such, there is a growing need to evaluate how different mRNA modalities perform with clinically relevant delivery platforms. Although linRNA, circRNA, and saRNA have diverged through targeted engineering, they share a common origin in early mRNA technologies and rely on efficient intracellular delivery for protein expression. Delivery systems have similarly progressed, from early polycations like polyethyleneimine to clinically validated lipid nanoparticles (LNPs) and emerging bioreducible polymers such as pABOL.^[^ ^30^, ^41^, ^42^ ^]^ Previous studies have compared RNA modalities in isolation or under variable conditions. For example, Qu et al. (2022) compared circRNA and linRNA delivered via LNPs;^[^ ^43^ ^]^ Vogel et al. (2018) showed saRNA could achieve equivalent immunity to linRNA at lower doses when delivered with LNPs;^[^ ^44^ ^]^ polymer‐based delivery of saRNA has been studied, while charge‐altering releasable transporters have been used to deliver circRNA to elicit antigen‐specific responses and enable sustained in vivo protein production.^[^ ^19^, ^30^, ^45^ ^]^ However, no study to date has directly compared linRNA, circRNA, and saRNA using both LNP and polymeric platforms under standardized conditions. To address this, we systematically evaluated linRNA, circRNA, and saRNA delivered via LNPs and pABOL PNPs, focusing on the interplay between transcript properties, delivery and protein expression kinetics in vivo.

Consistent with prior findings,^[^ ^44^ ^]^ administration of the VEEV‐based saRNA resulted in significantly higher protein expression than non‐replicating mRNA. While all mRNA constructs encoded the same firefly luciferase gene, differences in expression magnitude were influenced by transcript‐specific features, particularly the UTRs flanking the coding sequence. The distinct UTRs in the non‐replicating mRNAs likely modulated transcript stability and translation efficiency, contributing to variation relative to the standardized viral elements in saRNA. Impressively, sub‐microgram administration of VEEV‐saRNA using pABOL or LNP resulted in higher expression than 5 µg of non‐replicating mRNA. Molar calculations reveal that 0.5 µg of saRNA (∼9.3 × 10¹⁰ molecules) yields ∼30–50 times fewer transcripts than 5 µg of linRNA (∼4–5 × 10¹^2^ molecules), yet still outperforms them in protein expression. This study and others have demonstrated that saRNA can be administered at significantly lower doses than non‐replicating mRNA, especially when considered on a per‐transcript level, because saRNA is ∼3 times larger than conventional mRNA.^[^ ^46^, ^47^ ^]^ Vogel et al. showed that VEEV‐saRNA luciferase expression in mice lasted ten more days above the peak level of linRNA. Additionally, it was shown that a 64‐fold lower dose of saRNA than mRNA was required to protect mice challenged with the influenza virus.^[^ ^44^ ^]^ LNP delivery of linRNA^BNT^ resulted in higher expression than linRNA^TM^ ^V^ thus demonstrating that further optimization of the non‐coding elements of mRNA transcripts would result in higher translation of encoded therapeutic proteins. The AES‐mtRNR1 3′ UTR present in linRNA^BNT^ was discovered using systematic evolution of ligands by exponential enrichment in a study attempting to replace the use of alphaviral saRNA vectors in generating clinical‐grade induced pluripotent stem cells.^[^ ^33^, ^48^, ^49^ ^]^ The optimized linRNA^BNT^ resulted in relatively similar total luciferase expression as circRNA, albeit slightly lower. Chen et al. showed that 10 µg of circRNA encoding human erythropoietin (hEPO) resulted in durable plasma levels of hEPO 96 h after administration, compared to linRNA which declined to baseline levels by 48 h.^[^ ^19^ ^]^ At 5 µg in our study, we observed luciferase expression of circRNA and linRNA^BNT^ for up to 22 and 16 days, respectively. Although they have slightly similar half‐lives in mammalian cells (3 and 4 h for luciferase and hEPO, respectively), systemic clearance and renal filtration may shorten the observed duration of hEPO protein levels.^[^ ^50^, ^51^ ^]^ Additionally, the variation in duration of expression observed could be attributed to differences in route of administration (intraperitoneal versus intramuscular), delivery platform (lipid nanoparticles versus charge‐altering releasable transporters) and/or different quality control standards (commercially sourced versus in‐lab production).

Substantial interplay between the delivery platform and protein expression has been reported for saRNA and linRNA.^[^ ^26^, ^52^ ^]^ In our study, intramuscular delivery of saRNA, linRNA and circRNA using LNPs resulted in expected differences in duration and magnitude of luciferase expression, consistent with their respective transcript architectures. In contrast, pABOL delivery of non‐replicating mRNA resulted in subpar expression compared to the higher expression of saRNA PNPs, possibly due to the length difference between linRNA, circRNA and saRNA. Blakney et al. noted that the efficient delivery of large nucleic acid molecules, such as plasmid DNA and saRNA, required large molecular weight copolymers with high charge density, whereas smaller mRNA transcripts benefited from lower molecular weight polymers with low charge density.^[^ ^53^ ^]^ Since polymer‐based delivery systems must be tailored to match the physicochemical requirements of the nucleic acid cargo, one key variable in our study was the mRNA size. The VEEV‐saRNA used in this study (9488 nucleotides) was 3–5 times longer than the linRNA (1767–2085 nt) or circRNA (2570 nt). pABOL, as used in this study, was initially optimized for saRNA delivery and may therefore have been suboptimal for linRNA or circRNA. Systematic tuning of the polymer's molecular weight, cationic block length, and buffering capacity could improve the delivery of smaller RNAs.

Interestingly, previous studies have reported that pABOL delivery of saRNA resulted in 10‐100‐fold higher saRNA expression than LNPs compared to the 2‐fold difference we observed. Bathula et al. reported a 10‐fold difference in saRNA expression after pABOL delivery compared to LNPs.^[^ ^26^ ^]^ Similar parameters for formulating LNPs and PNPs were used between our study and the study reported by Bathula et al., but differed in the generation of the saRNA transcripts. Our study utilized an optimized VEEV‐saRNA vector with a longer poly‐A tail and a single guanosine nucleoside added at the +1 site of the saRNA transcript compared to the standard VEEV saRNA transcript with a 30‐nucleotide poly‐A tail and 2 guanosines preceding the 5′ UTR.^[^ ^37^ ^]^ The additional guanosines at the +1 site of saRNA transcripts are an artefact of using a standard T7 RNA polymerase promoter sequence. Alphaviruses have a conserved 5′ terminal AU dinucleotide that is preferentially recognized by non‐structural protein 1, with implications for capping efficiency and transcript replication.^[^ ^54^ ^]^ The high in vivo bioactivity of the optimized saRNA vector may have minimized the observed differences in transgene output between LNP and paBOL delivery. Similarly, Blakney and colleagues reported that pABOL‐saRNA formulations resulted in 7‐ to 63‐fold higher intramuscular expression than LNPs at day 7.^[^ ^30^ ^]^ Notably, Blakney et al. used a saRNA derived from the Trinidad donkey variant of the VEEV genus, while this study utilized the TC‐83 variant that is reported to result in higher protein expression.^[^ ^23^ ^]^ Additionally, the comparison in Blakney et al. used LNP formulations with a different proprietary ionizable lipid and an N/P ratio of 8, whereas in this study, an N/P ratio of 10 and the clinically utilized ALC‐0315 lipid were used. ALC‐0315 is a branched, ionizable lipid with biodegradable ester linkages, and has been shown to enhance the delivery of encapsulated nucleic acids.^[^ ^55^ ^]^ The elevated LNP‐saRNA performance in our study likely contributed to narrowing the gap that has historically favored pABOL‐saRNA in other settings, while subtle differences in polymer batch characteristics, such as molecular weight distribution or formulation reproducibility, may further complicate cross‐study comparisons.

Clinical assessment of the Kostaive VEEV‐saRNA vaccine delivered in LNPs resulted in higher immunogenicity compared the Comirnaty linRNA vaccine against SARS‐CoV‐2.^[^ ^4^ ^]^ Due to the increased interest in saRNA therapies, other groups are also using the VEEV‐based vector for immunotherapy,^[^ ^56^ ^]^ protein replacement^[^ ^6^ ^]^ and induction of pluripotency.^[^ ^49^ ^]^ Ubiquitous application of the VEEV saRNA vector across diverse therapeutic areas maybe suboptimal, as its rapid replication kinetics and strong host cell interactions may not align with the expression requirements or safety profiles needed for certain applications.^[^ ^5^ ^]^ For instance, protein replacement therapies using saRNA may require repeat administration to maintain therapeutics levels, which could lead to build up of adaptive immunity against the VEEV‐derived replicase proteins.^[^ ^6^ ^]^ Such immune responses may attenuate efficacy over time or introduce safety concerns, particularly for chronic or regenerative applications. These considerations underscore not only the importance of aligning saRNA vector properties with therapeutic goals, but also the need to develop and characterize genotypically distinct saRNA vectors that may be better suited to different clinical contexts. Our results and others’ have shown that New and Old World alphaviruses can be adapted into saRNA vectors with different pharmacokinetics, owing to their differential interactions with host cell machinery.^[^ ^23^, ^57^, ^58^, ^59^ ^]^ LNP and pABOL delivery of saRNA result in different levels of transfection efficiency as well as local and systemic inflammation thus creating an environment that augments or ablates expression of New or Old World alphaviruses. Dominguez et al. reported that EEEV and VEEV saRNAs produced more heterologous proteins than CHIKV and SINV saRNAs owing to the latter saRNAs causing high levels of host cell translation and transcriptional inhibition.^[^ ^58^ ^]^ Consistently, LNP delivery of New World alphaviral saRNA, i.e., EEEV, WEEV and MADV resulted in higher luciferase expression than CHIKV‐ and SINV‐based saRNA vectors. No substantial difference was observed between the New and Old World alphaviral‐saRNA upon pABOL delivery. It is possible that lower transfection in the muscle tissue and limited cellular tropism by pABOL‐saRNA would curtail the expected differences between the saRNA vectors.^[^ ^26^ ^]^ Currently it is unknown if pABOL‐saRNA polyplexes transfect specific cell types after intramuscular administration. On the other hand, Kimura et al. observed saRNA expression in myocytes and infiltrating mononuclear cells after intramuscular injection of LNPs formulated with ALC‐0315 as the ionizable lipid.^[^ ^25^ ^]^ Collectively, these findings highlight that the magnitude and duration of saRNA‐driven protein expression are not solely determined by the RNA sequence, but rather emerge from a complex interplay between vector genotype, delivery system, and target tissue biology. Future studies should incorporate both transcript design and delivery efficiency into the development pipeline to maximize therapeutic output.

As interest grows in applying mRNA‐based platforms for protein replacement therapies, it is critical to consider not only expression kinetics but also the molecular compatibility between the mRNA modality and the host's protein processing machinery. The innate immune response elicited by both the mRNA modality and its delivery vehicle further shapes the intracellular environment for protein maturation and proper post‐translational modification (PTM).^[^ ^60^, ^61^ ^]^ LNPs, while highly efficient, are known to induce acute inflammatory responses that can alter endoplasmic reticulum (ER) function and secretory pathway dynamics.^[^ ^25^, ^30^, ^61^ ^]^ Conversely, pABOL elicits lower levels of innate immune signaling, potentially offering a more permissive setting for high‐fidelity protein expression.^[^ ^26^ ^]^ On the other hand, the intrinsic properties of the mRNA modalities such as dsRNA content, capping efficiency (in linRNA and saRNA) and the use of modified or unmodified nucleotides can activate cytosolic and endosomal sensors leading to interferon signaling and ER stress.^[^ ^62^, ^63^ ^]^ saRNA, due to replication‐associated double‐stranded RNA intermediates, typically triggers stronger antiviral responses, which may suppress translation and downregulate PTM‐related enzymes.^[^ ^5^, ^60^, ^64^ ^]^ Additionally, the activities of the non‐structural protein of saRNA vectors contribute to the complexity of this interplay. The replicase components disrupt host cell pathways involved in protein folding, trafficking, and secretion. For example, CHIKV nsP2 induces ER stress and inhibits host transcription, while SINV nsP3 sequesters G3BP proteins, disrupting autophagy and stress granule dynamics, both of which can hinder proper folding and impair secretion or PTM such as glycosylation or furin‐mediated cleavage of complex therapeutic proteins.^[^ ^65^, ^66^ ^]^ While we show these trends for luciferase expression after LNP and pABOL delivery of linRNA, circRNA and saRNA, we anticipate further differences caused by variation in the encoded therapeutic gene in addition to the vector and delivery formulation. Therefore, effective mRNA‐based protein replacement requires integrated optimization of RNA design, delivery chemistry, and immunological context to ensure both robust expression and proper protein maturation.

## 4 Conclusion

This study highlighted how both mRNA modality and delivery platform shape in vivo expression kinetics, but several limitations should be acknowledged. All delivery experiments were performed via intramuscular injection, and it remains uncertain whether the observed expression profiles would translate to other tissues, administration routes, or disease contexts.^[^ ^67^ ^]^ Although formulation parameters, such as dose, particle size, and N/P ratio, were standardized, batch‐to‐batch variability in polymer or LNP preparations may have affected delivery performance. Furthermore, we did not assess immune activation, biodistribution, or cellular tropism which are critical factors for evaluating therapeutic efficacy and safety.^[^ ^68^, ^69^ ^]^ Lastly, the use of firefly luciferase as a model protein, due to its favorable enzymatic profile such as sensitivity and signal amplification,^[^ ^70^ ^]^ limits its generalizability to more complex therapeutic proteins. Given the sensitivity and stability of luciferase, particularly in tissues with low protein turnover, a persistent signal may reflect detection saturation or residual reporter activity rather than ongoing transcript‐driven protein production. These properties can lead to overestimation of delivery efficiency and expression output compared to more complex therapeutic proteins that require substantial post‐translational modifications.^[^ ^71^ ^]^ Therefore, validation with clinically relevant therapeutic proteins will be necessary to assess the translatability of mRNA modalities and delivery platforms for therapeutic application.

Despite these limitations, our findings demonstrate that self‐amplifying RNA consistently achieved higher and more durable protein expression than non‐replicating mRNA, regardless of whether it was delivered in LNPs or PNPs. Differences in duration and magnitude of protein expression, influenced by both mRNA modality and delivery system, underscore the robustness and complexity of RNA therapeutics. These results encourage further optimization of mRNA modalities and delivery formulations to enhance therapeutic protein production. With further refinement, mRNA‐based therapies have the potential to transform treatment paradigms across a wide range of therapeutic and prophylactic applications.

## 5 Experimental Section

### Materials

[(4‐hydroxybutyl)azanediyl)]di(hexane‐6,1‐diyl) bis(2‐hexyldecanoate) (BroadPharm, ALC‐0315 #BP25498); 1,2‐dioleoyl‐*sn*‐glycero‐3‐phosphoethanolamine (Avanti Polar Lipids, DOPE #BP25709); plant‐derived cholesterol (Millipore Sigma #700100P, USA); 1,2‐dimyristoyl‐*rac*‐glycero‐3‐methoxypolyethyleneglycol‐2000 (Avanti Polar Lipids, DMG‐PEG 2000 #880 120); D‐Luciferin Substrate (Revvity #122 799, USA); N,N’‐Bis(acryloyl)cystamine (Millipore Sigma #A4929); 4‐amino‐1‐butanol (Millipore Sigma #178 330)

### Plasmids

For conventional non‐replicating mRNA, firefly luciferase (Fluc) encoding sequences were flanked by 5′ and 3′ UTR from Tobacco mosaic virus or sequences used in the Comirnaty vaccine.^[^ ^31^, ^34^ ^]^ saRNA vectors were constructed as previously described by replacing the structural protein sequences with Fluc using the NEBuilder HIFI DNA assembly.^[^ ^72^ ^]^ The GenBank number of the alphavirus sources were provided in Table S2 (Supporting Information). At the 5′ end, the saRNA or mRNA elements were flanked by T7 polymerase promoter while at the 3′ end they were flanked by poly(A) tail followed by SapI cleavage site. All plasmids were propagated in *Escherichia coli* (NEB), cultured in Luria Broth with 100 µg mL^−1^ carbenicillin or Kanamycin and extracted using a plasmid maxiprep kit (Qiagen, USA).

### RNA Synthesis

Circular RNA was prepared by GenScript. All linear mRNA and saRNA transcripts were prepared in the lab as follows: Plasmids were linearized using SapI for 2 h at 37 °C followed by inactivation at 60 °C for 20 min. 50 µg mL^−1^ of linearized plasmid was mixed with Tris‐HCl, magnesium acetate, spermidine, dithiothreitol, nucleotide triphosphates, pyrophosphatase, RNase inhibitor, and T7 RNA polymerase (NEB #M0251L) as previously described.^[^ ^73^ ^]^ Unmodified nucleotides were used for saRNA while 100% replacement of uridine with N1‐methylpseudouridine was done for conventional mRNA. In vitro *transcription* reactions for mRNA and saRNA were performed for 2 h at 3 and 30 °C, respectively, and further capped using the CellScript Cap‐1 system (CellScript #C‐SCCS1710, USA) according to manufacturer's instructions. All RNAs were purified by lithium chloride precipitation and dissolved in RNAse‐free water. The integrity of the RNA was quantified using capillary electrophoresis.

### Double Stranded RNA Quantification

RNA samples were prepared with 125 ng total RNA in 200 µL STE buffer (0.1 M NaCl, 1 mM EDTA, 50 mM Tris‐HCl pH 7.0). A standard control was created using a 500 bp dsRNA (UBC RNA Formulation Core #DR500NM), two‐fold serially diluted from 50 to 0.4 pg µL^−1^. Samples were then loaded onto a positively charged membrane (Cytiva #RPN1210B) pre‐soaked in Milli‐Q water using a vacuum filtration apparatus following the manufacturer's instructions (Bio‐Rad #1 706 542). The membrane was then incubated in blocking buffer (Bio‐Rad #12 010 020) at room temperature for 15 min with agitation. After blocking, it was incubated with J2 anti‐dsRNA murine antibody (Scicons #10 010 500), diluted 1:5000 in blocking buffer, at 4 °C overnight with agitation. Following antibody incubation, the membrane was washed three times with TBS‐T (0.1% v/v Tween‐20, 150 mM NaCl, 20 mM Tris‐HCl pH 7.5) at room temperature for 15 min per wash with agitation. Next, the membrane was incubated with Alexa Fluor 680‐conjugated anti‐mouse goat antibody (Invitrogen #A21057), diluted 1:10 000 in TBS‐T, at room temperature for 1 h with agitation. This was followed by three additional washes as described above. Fluorescence detection was performed using the Sapphire Biomolecular Imager (Azure Biosystems, USA). Signal intensities were quantified using ImageJ, and a four‐parameter logistic sigmoidal curve was fitted to the dsRNA standard data in GraphPad Prism. This standard curve was used to determine the dsRNA content in each RNA construct.

### Formulating mRNA‐lipid Nanoparticles

Lipid nanoparticles encapsulating the various mRNA modalities were formulated as previously optimized.^[^ ^38^ ^]^ Lipids were dissolved in ethanol at molar ratios of 45:15.9: 37.85:1.25 (ALC‐0315: DOPE: Cholesterol: DMG‐PEG). The aqueous phase containing the mRNAs at 0.127 mg mL^−1^ was prepared in in 25 mM sodium acetate buffer at pH 4.5. At an N/P ratio of 10, the aqueous and lipid phases were rapidly mixed in a T‐junction mixer using the Havard Apparatus 33 DDS Syringe pump (Darwin Microfluidics) at a flow rate of 15 and 5 mL per minute, respectively. LNPs were diluted 10‐fold with PBS and dialyzed using sterilized MWCO 10 kDa centrifugal filters (Amicon, Millipore Sigma) at 4 °C and 2000 × g. As previously described, the Quant‐iT Ribogreen RNA assay kit (ThermoFisher Scientific) was used to determine encapsulation efficiency and RNA concentrations in the LNPs.^[^ ^74^ ^]^

### Formulating mRNA‐pABOL Nanoparticles

pABOL was synthesized as previously described.^[^ ^26^, ^28^ ^]^ Briefly, N,N’‐Bis(acryloyl)cystamine (221 mg), 4‐amino‐1‐butanol (78 µL) and triethylamine (12 µL) were dissolved in a 4:1 methanol/water mixture (334 µL total volume) and stirred with a magnetic stir bar at 60 °C for 48 h. The reaction was then quenched by adding a 10% molar excess of 4‐amino‐1‐butanol and incubate for an additional 12 h at 60 °C. The resulting viscous mixture was diluted with 50 mL of methanol, and the pH adjusted to 4.0 using 1 M hydrochloric acid. The polymer solution was dialyzed against acidified water (pH 4.0), replacing the dialysate twice daily for three days. The dialyzed solution was lyophilized to yield pABOL as a white power, which was stored under argon at −70 °C until formulation. On the day of use, pABOL powder was dissolved in RNAse‐free water at 50 mg mL^−1^ while the mRNAs were diluted to 0.125 mg mL^−1^ in HEPES buffer (pH 5.8, 5% glucose, 20 mM HEPES). pABOL and mRNA polyplexes were mixed at weight‐to‐weight ratio of 45:1. 400 µL of RNA solution was titrated into 100 µL of pABOL solution at a rate of 160 µL min^−1^ using the Havard Apparatus 33 DDS Syringe pump.

### Characterizing Physical Properties of Nanoparticles

Size, polydispersity index, and zeta potential of the LNPs were measured using the Zetasizer Nano (Malvern Instruments) and Zetasizer 7.12 software (Malvern). LNPs and PNPs were diluted 10‐fold in PBS or HEPES, respectively.

### In Vivo Bioluminescence Imaging

All animal studies were approved by the University of British Columba Animal Care Committee and complied to all ethical regulations for animal testing and research. Female BALB/c mice (Jackson Laboratories, USA) at 6–8 weeks received bilateral intramuscular injections in the hind limbs. 0.5 or 50 µg of mRNA in LNPs or PNPs were administered. On specific days as indicated in the results section, mice were imaged on the IVIS Lumina LT (Revvity, USA) using a 3 min exposure after receiving 100 µL of 30 mg mL^−1^ D‐Luciferin Substrate (Revvity) and allowed to rest for 7 min. Whole body flux (p/s) was quantified using the Living Imaging Software (Revvity).

### Statistical Analysis

Statistical analysis was performed in GraphPad Prism, version 10.3.0, and was reported for each figure in the corresponding figure legend. Figure 1A–C were created with Biorender.com.

## Conflict of Interest

S.J.M., P.A, N.S.W., and A.J.G. are employees of Replicate Bioscience. A.K.B. and I.C.C. are co‐inventors on saRNA‐related patents. A.K.B. serves on the scientific advisory board for Replicate Biosciences, Genvax Technology, and Pasture Biosciences. Other authors declare no conflict of interests.

## Supporting information



Supporting Information

## Data Availability

The data that support the findings of this study are available from the corresponding author upon reasonable request.
