# Supplementary material for: Divergent Delivery and Expression Kinetics of Lipid and Polymeric Nanoparticles across mRNA Modalities
Source: Adv Sci (Weinh). 2025 Jul 12;12(38):e08907. doi: 10.1002/advs.202508907 (PMC12520532; doi:10.1002/advs.202508907)
Supplement: Supplementary file 1 — Supporting Information [file ADVS-12-e08907-s001.pdf]

ADVANCED SCIENCE
Open Access

Supporting Information

for *Adv. Sci.*, DOI 10.1002/advs.202508907

Divergent Delivery and Expression Kinetics of Lipid and Polymeric Nanoparticles across mRNA Modalities

*Irafasha C. Casmil, Josh J. Friesen, Nuthan V. Bathula, Anneke Strumpel, Chia Hao Ho, Ilana Guez, Kristen Y. S. Kong, Andrew J. Varley, Shigeki J. Miyake-Stoner, Parinaz Aliahmad, Nathaniel S. Wang, Andrew J. Geall and Anna K. Blakney**

Divergent Delivery and Expression Kinetics of Lipid and Polymeric Nanoparticles Across mRNA Modalities

*Irafasha C. Casmil, Josh J. Friesen, Nuthan V. Bathula, Anneke Strumpel, Chia Hao Ho, Ilana Guez, Kristen Y.S. Kong, Shigeki J. Miyake-Stoner, Parinaz Aliahmad, Nathaniel S. Wang, Andrew J. Geall, Andrew J. Varley, Anna K. Blakney**

*Email: anna.blakney@msl.ubc.ca

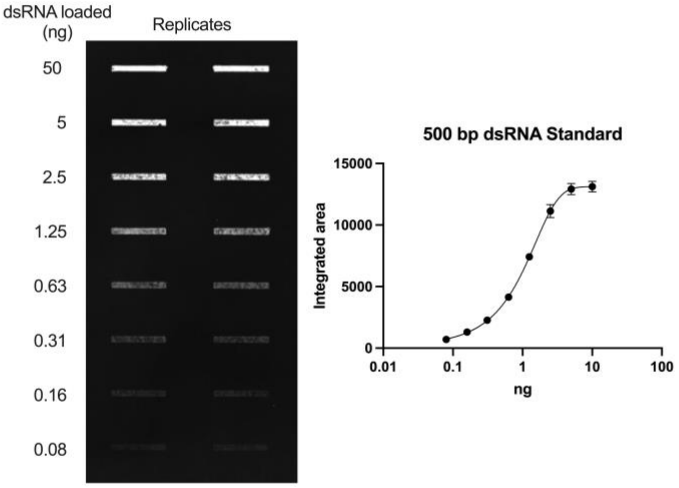

Figure S1. Dot blot image of double stranded RNA standard and standard curve of signal intensities (R^2 = 0.9982) as quantified using ImageJ.

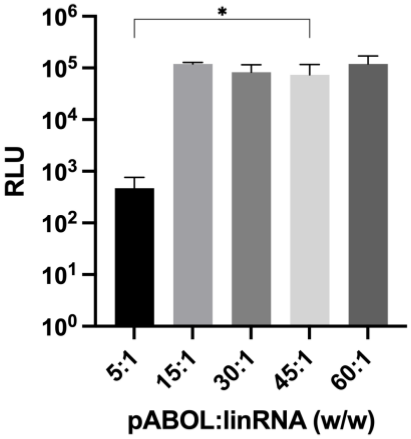

Figure S2. Luciferase activity in HEK 293T cells. 50,000 HEK cells per well were seeded overnight in complete DMEM media (10% fetal bovine serum and 1% Pen-Strep). Various pABOL:linRNA (w/w) ratio were used to generate polymeric nanoparticles and added to the wells.

24 hours after transfection into HEK cells, luciferase assay was performed. Data shown are mean values ± standard deviation from n = 3 replicates compared to the standard 45:1 ratio using One-way ANOVA test.

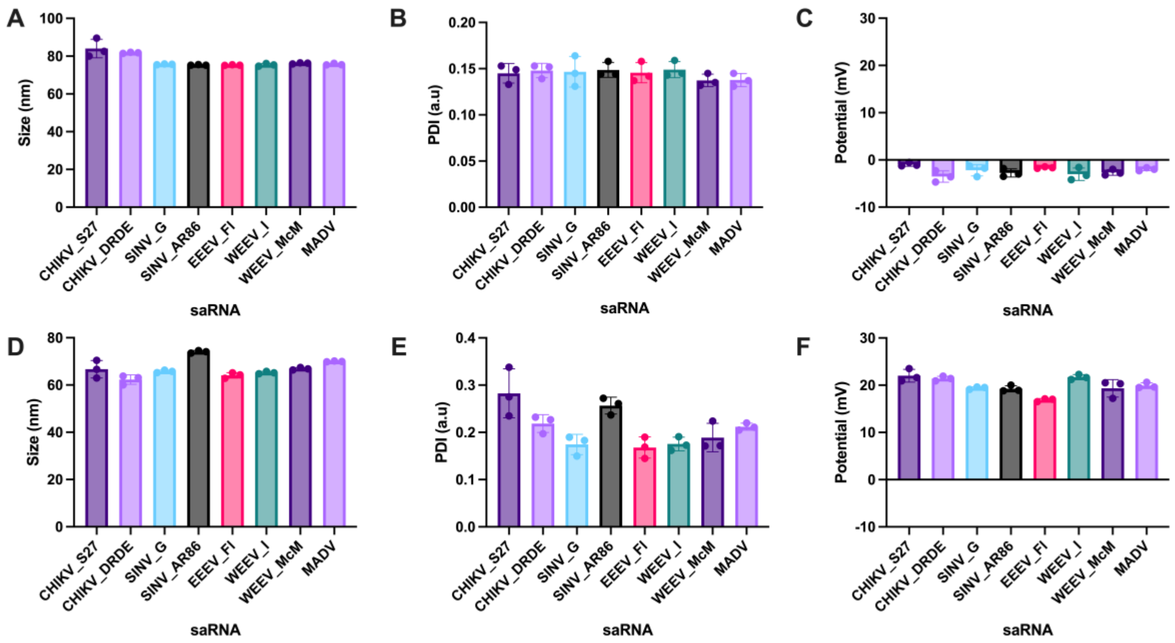

Figures S3. Physicochemical properties of saRNA nanoparticles. LNP-saRNA average (A) zeta size in nanometers, (B) polydispersity index and (C) zeta potential in millivolts. pABOL-saRNA average (D) zeta size in nanometers, (E) polydispersity index and (F) zeta potential in millivolts of LNPs. Data shown are mean values ± standard deviation from n = 3 technical replicates compared using One-way ANOVA followed by Tukey's multiple comparison test.

Table S1. Source of alphaviral sequences adapted into saRNA vectors

saRNA	GenBank
Venezuelan equine encephalitis virus TC83 strain	L01443
Chikungunya virus S27 strain	AF369024
Chikungunya virus DRDE-06 strain	EF210157
Sindbis virus Girdwood strain	MF459683
Sindbis virus AR86 strain	U38305
Eastern equine encephalitis virus Florida strain	EF151502
Western equine encephalitis virus Imperial strain	GQ287641
Western equine encephalitis virus McMillan strain	GQ287640
Madariaga virus	KJ469641

Table S2. Estimated moles of saRNA transcripts per 5 μg dose

saRNA	Length[a]	Moles[b] ($\times10^{-12}$)
Venezuelan equine encephalitis virus TC83 strain	9488	1.64
Chikungunya virus S27 strain	9876	1.57
Chikungunya virus DRDE-06 strain	9872	1.58
Sindbis virus Girdwood strain	9880	1.57
Sindbis virus AR86 strain	9846	1.58
Eastern equine encephalitis virus Florida strain	9749	1.60
Western equine encephalitis virus Imperial strain	9614	1.62
Western equine encephalitis virus McMillan strain	9602	1.62
Madariaga virus	9674	1.61

[a)]Length of saRNA in nucleotides from the from the first nucleotide to the last adenosine in the poly-A tail; [b)]Moles of RNA calculated using equation 1 and assuming 321.47 g/mol as the average molecular weight of a nucleotide.

Equation 1 $$\text{Moles} = \frac{\text{Mass (g)}}{\text{length} \times 321.47\frac{\text{g}}{\text{mol}}}$$
